# p300-mediated acetylation increased the protein stability of HIPK2 and enhanced its tumor suppressor function

**DOI:** 10.1038/s41598-017-16489-w

**Published:** 2017-11-23

**Authors:** Jong-Ryoul Choi, Seo-Young Lee, Ki Soon Shin, Cheol Yong Choi, Shin Jung Kang

**Affiliations:** 1 Department of Molecular Biology, Sejong University, Seoul, 05006 Republic of Korea; 2 Department of Biology, Kyung Hee University, Seoul, 02447 Republic of Korea; 3 Department of Life and Nanopharmaceutical Sciences, Kyung Hee University, Seoul, 02447 Republic of Korea; 4 Department of Biological Sciences, Sungkyunkwan University, 2066 Seobu-ro, Suwon, 16419 Republic of Korea; 5 Department of Integrative Bioscience and Biotechnology, Sejong University, Seoul, 05006 Republic of Korea; 6 Present Address: Massachusetts General Hospital, Cutaneous Biology Research Center, Building 149, 13th Street, Charlestown, MA 02129 USA

## Abstract

Homeodomain-interacting protein kinase 2 (HIPK2) is a nuclear serine/threonine kinase that functions in development and tumor suppression. One of the prominent features of this kinase is that it is tightly regulated by proteasomal degradation. In the present study, we present evidence suggesting that the protein stability of HIPK2 can be regulated by p300-mediated acetylation. p300 increased the protein level of HIPK2 via its acetyltransferase activity. p300 increased the acetylation of HIPK2 while decreased polyubiquitination and its proteasomal degradation. We also observed that DNA damage induced acetylation of HIPK2 along with an increase in the protein amount, which was inhibited by p300 RNAi. Importantly, p300 promoted p53 activation and the HIPK2-mediated suppression of cell proliferation, suggesting acetylation-induced HIPK2 stabilization contributed to the enhanced activation of HIPK2. Overexpression of p300 promoted the HIPK2-mediated suppression of tumor growth in mouse xenograft model as well. Taken together, our data suggest that p300-mediated acetylation of HIPK2 increases the protein stability of HIPK2 and enhances its tumor suppressor function.

## Introduction

Homeodomain-interacting protein kinase 2 (HIPK2) is a nuclear serine/threonine kinase that regulates development and cell death by phosphorylating various transcription factors and regulators of apoptosis^1^. HIPK2 promotes apoptosis following lethal DNA damage by phosphorylating and thus stabilizing p53 to activate transcription of proapoptotic genes^2,3^. HIPK2 can induce apoptosis also in a p53-independent manner by phosphorylating the transcriptional corepressor carboxyl-terminal binding protein after UV irradiation^4^. Recently, it has been reported that HIPK2 can control the cellular survival threshold in response to oxidative stress^5^. Accumulating evidence suggests that HIPK2 is a regulator of cell fate between survival and death under cellular stress conditions. Therefore, it is necessary to regulate the activation of HIPK2 tightly for the proper cellular response to various stressors.

The activity of HIPK2 can be regulated by proteolysis or altering subcellular localization^6–8^. HIPK2 is normally localized in the nuclear speckles but can be sequestered to cytoplasm by Src kinase^6^ or oncogenic PEBP-2b-SMMHC chimeric protein^7^. More generally, HIPK2 activity is kept low under normal condition by constant proteasomal degradation^8^. It has been reported that proteasomal degradation of HIPK2 is mediated by ubiquitin ligases such as seven in absentia homolog (Siah)1, Siah2, WD40 repeat/SOCS box-containing protein1 (WSB1), and Skp1/Cullin1/Fbx3 complex (SCF^Fbx3^)^9–12^. Siah1, WSB1, and SCF^Fbx3^ have been shown to promote ubiquitination of HIPK2 in unstressed cells whereas Siah2-mediated ubiquitination of HIPK2 is much more prominent under hypoxic conditions^9–12^. The involvement of multiple ubiquitin ligases for the degradation of HIPK2 in different cellular conditions suggests that regulation of HIPK2 activation is of great importance in managing response to cellular stressors such as DNA damage and hypoxia.

Previously we showed that a mammalian homolog of silent information regulator 2, Sirt1 deacetylase, promotes deacetylation of HIPK2 and its subsequent ubiquitination and degradation^13^. The regulation of ubiquitin-mediated proteasomal degradation by acetylation or deacetylation is not uncommon. For example, acetylation of tau is reciprocally regulated by acetyltransferase p300 and deacetylase Sirt1^14^. When tau is deacetylated by Sirt1, ubiquitination is promoted for the proteasomal degradation^14^. A tumor suppressor liver kinase B1 (LKB1) is also deacetylated by SIRT1 and then ubiquitinated for the proteasomal degradation^15^. Similar modulation of proteasomal degradation by acetylation/deacetylation has been shown in the case of p53, Foxp3, and Smad7^16–18^. Because both acetylation and ubiquitination target lysine residues, it is possible that acetyltransferases/deacetylases and ubiquitin ligases compete for the same lysine residues on the substrate proteins.

Since we observed that ubiquitination of HIPK2 can be modulated by Sirt1-mediated deacetylation^13^, we reasoned that there may be an acetyltransferase that can acetylate HIPK2 to block ubiquitination. A previous study reported that HIPK2 interacts and phosphorylates an acetyltransferase p300^19^. In the present study, we examined whether p300-induced acetylation of HIPK2 modulates its proteasomal degradation. We present evidence that HIPK2 acetylation induced by p300 can suppress ubiquitination and subsequent proteasomal degradation of HIPK2, which enhances the proapoptotic function of HIPK2.

## Results

### HIPK2 colocalized and interacted with p300

**Figure 1:**
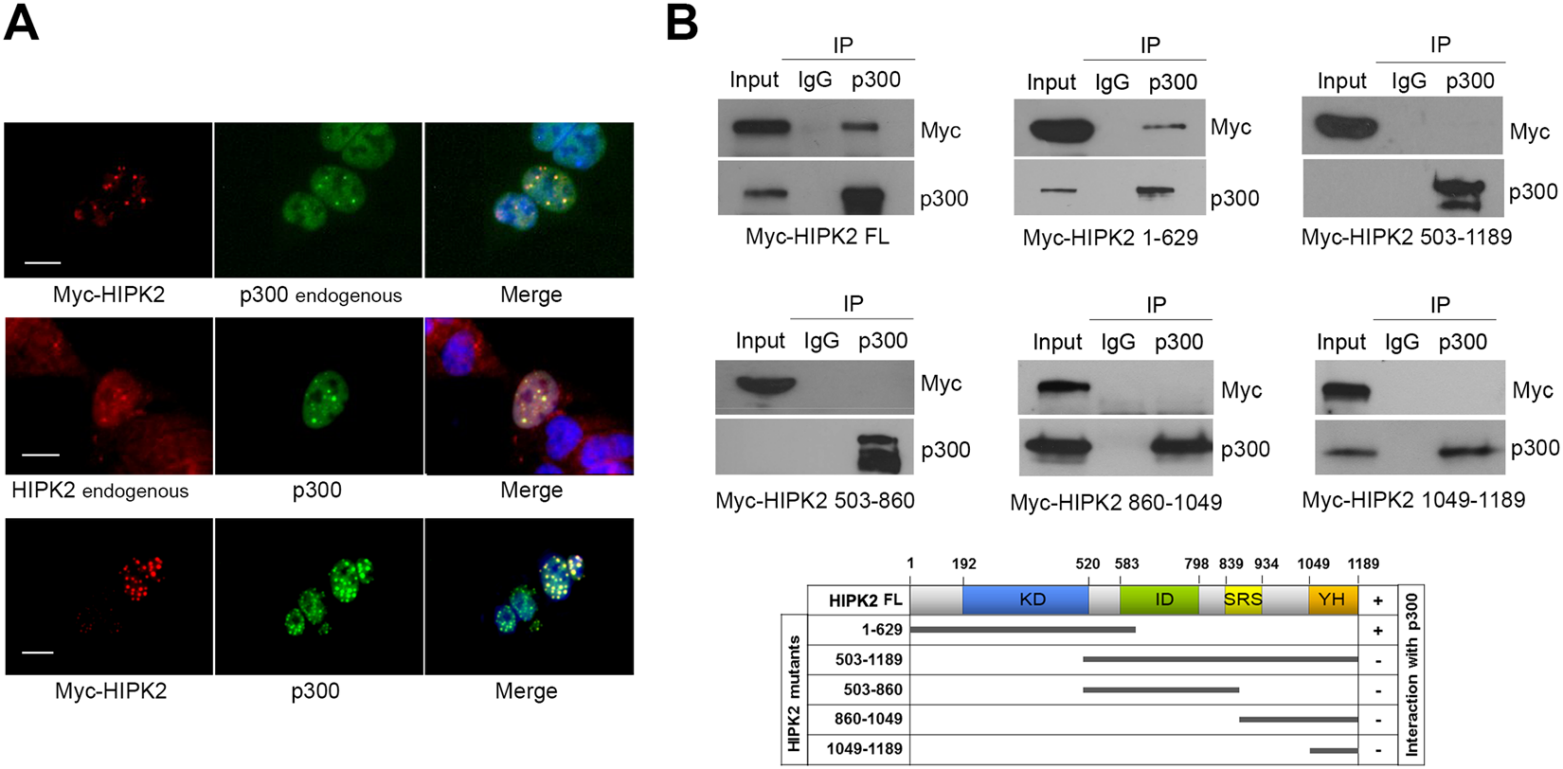
HIPK2 interacted with p300 in the nuclear speckles. (**A**) HEK293 cells were transfected with myc-HIPK2 or p300 expression plasmids and then immunostained using anti-myc, anti-HIPK2 or anti-p300 antibodies as indicated. Scale bar denotes 10 μm. (**B**) HEK293 cells were transfected with full-length (FL) or various truncated mutants of HIPK2 (amino acids 1-629, 503-1189, 503-860, 860-1049, and 1049-1189) together with p300 expression vectors. Then the cell lysates were immunoprecipitated using anti-p300 and then probed with anti-myc antibody to examine the interaction between p300 and HIPK2. Note the HIPK2 of full-length and N-terminal half (1-629) coimmunoprecipitated with p300 as depicted in a diagram (bottom).

A previous study showed a direct interaction between HIPK2 and p300 using coimmunoprecipitation and GST pull-down assay^19^. When we examined the subcellular localization of HIPK2 and p300 by immunostaining, HIPK2 was mostly confined in the nuclear speckles whereas p300 was localized in both speckles and nucleoplasm (Fig. 1A). The overexpressed HIPK2 colocalized with the endogenous p300 and the endogenous HIPK2, with the overexpressed p300 in the nuclear speckles. This result suggests that HIPK2 may interact with p300 in the nuclear speckles.

It was previously reported that p300 interacts with HIPK2 via its histone acetyltransferase (HAT) domain^19^. However, it remains unknown which domain of HIPK2 interacts with p300. To identify the p300-interacting regions in HIPK2, various deletion mutants of HIPK2 were overexpressed together with p300 and the interaction was examined by coimmunoprecipitation assay. As shown in Fig. 1B, amino terminal half of the HIPK2 containing the kinase domain (amino acids 1–629) coimmunoprecipitated with p300. However, the carboxy terminal half of HIPK2 (503–1189), interaction domain (amino acids 503–860), amino acids 860–1149 containing speckle retention signal, or amino acids 1149–1189 did not coimmunoprecipitate with p300 (Fig. 1B). These results suggest that HIPK2 interacts with p300 via its kinase domain-containing amino terminal half.

### p300 increased the protein expression of HIPK2 via its acetyltransferase activity

**Figure 2:**
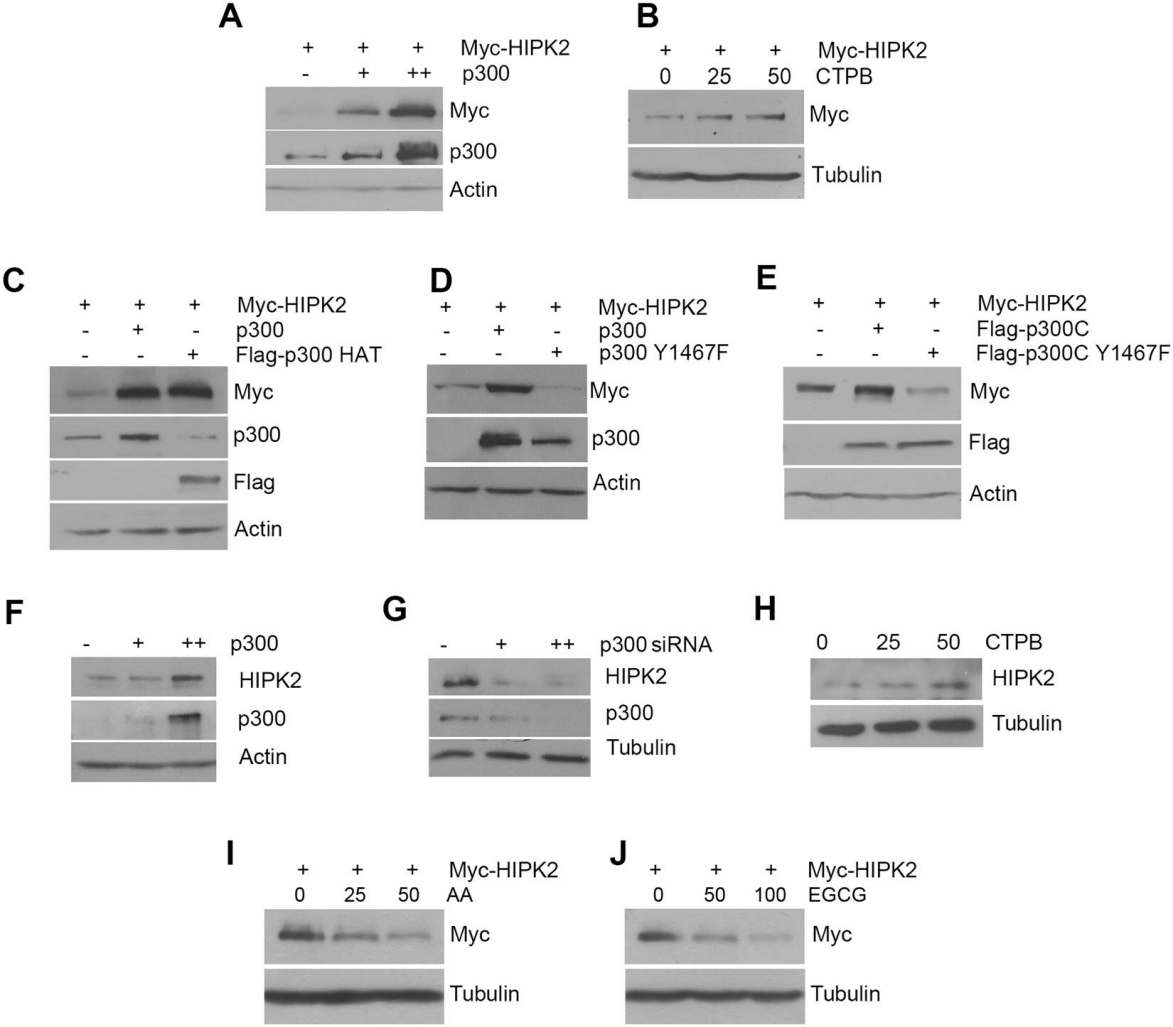
p300 increased the protein expression of HIPK2 via its acetyltransferase activity. (**A**) To examine the effect of p300 on the expression level of HIPK2, HEK293 cells were transfected with myc-HIPK2 with increasing amounts of p300 expression vectors (0, 0.25, 0.5 μg/ml) and then the protein amount of HIPK2 was examined by immunoblot using anti-myc antibody. (**B**) HEK293 cells were transfected with expression plasmid for myc-HIPK2 and then the cells were incubated in the presence of a p300 activator CTPB (0, 25, 50 μM) for 12 h. The cell lysates were processed for immunoblot using anti-myc antibody. (**C**) Full-length p300 or HAT domain only (HAT) was overexpressed together with myc-HIPK2 in the HEK293 cells and the protein level of myc-HIPK2 was examined by immunoblot. (**D** and **E**) Active-site mutant of full-length (d, p300 Y1467F) or C-terminal half containing the HAT domain (**E**, p300C Y1467F) was cotransfected with myc-HIPK2 expression vectors and then the protein expression of myc-HIPK2 was examined by immunoblotting. (**F–H**) To examine the effect of p300 on the protein level of the endogenous HIPK2, p300 was overexpressd (**F**), knocked down (**G**) or activated by CTPB (**H**; 0, 25, 50 μM) in HEK293 cells and the protein amount of endogenous HIPK2 was monitored. (**I** and **J**) To examine the effect of p300 inhibition, myc-HIPK2-overexpressing 293 cells were incubated with anacardic acid (AA) (G; 0, 25, 50 μM) or EGCG (H; 0, 50, 100 μM) for 12 h and then processed for anti-myc immunoblot. All the immunoblots were reprobed for actin or tubulin as indicated for loading controls.

Previously we showed that the proteasomal degradation of HIPK2 is promoted by SIRT1-mediated deacetylation^13^. In addition, we confirmed the interaction between p300 acetyltransferase and HIPK2 (Fig. 1). Thus we asked whether p300 can regulate the protein stability of HIPK2 possibly by acetylating HIPK2, counteracting the effect of SIRT1. To address this, HEK293 cells were transfected with expression vectors for myc-HIPK2 with or without p300 and then the expression level of myc-HIPK2 was examined. Overexpression of p300 dramatically increased the protein amount of the exogenous HIPK2 (Fig. 2A). To examine whether p300 increases HIPK2 expression via its enzymatic activity, the effect of p300 activator on the HIPK2 protein amount was examined. An activator of p300 CTPB increased the protein amount of HIPK2 (Fig. 2B). In addition, overexpression of the truncated mutant carrying only HAT domain of p300 increased the HIPK2 protein amount (Fig. 2C). Introduction of point mutation in the active site in the full-length p300 (Fig. 2D) or C-terminal half containing the HAT domain (Fig. 2E)^20^ abrogated the effect of p300 on HIPK2 expression. Importantly, endogenous HIPK2 protein level was altered in the same manner by overexpression (Fig. 2F) or knockdown (Fig. 2G) of p300 or by an activator (Fig. 2H). Furthermore, inhibitors of p300 such as anacardic acid and EGCG decreased the expression of exogenous HIPK2 (Fig. 2I and J). These results suggest that p300 increased the protein expression of HIPK2 via its acetyltransferase activity.

### p300 increased the protein stability of HIPK2

**Figure 3:**
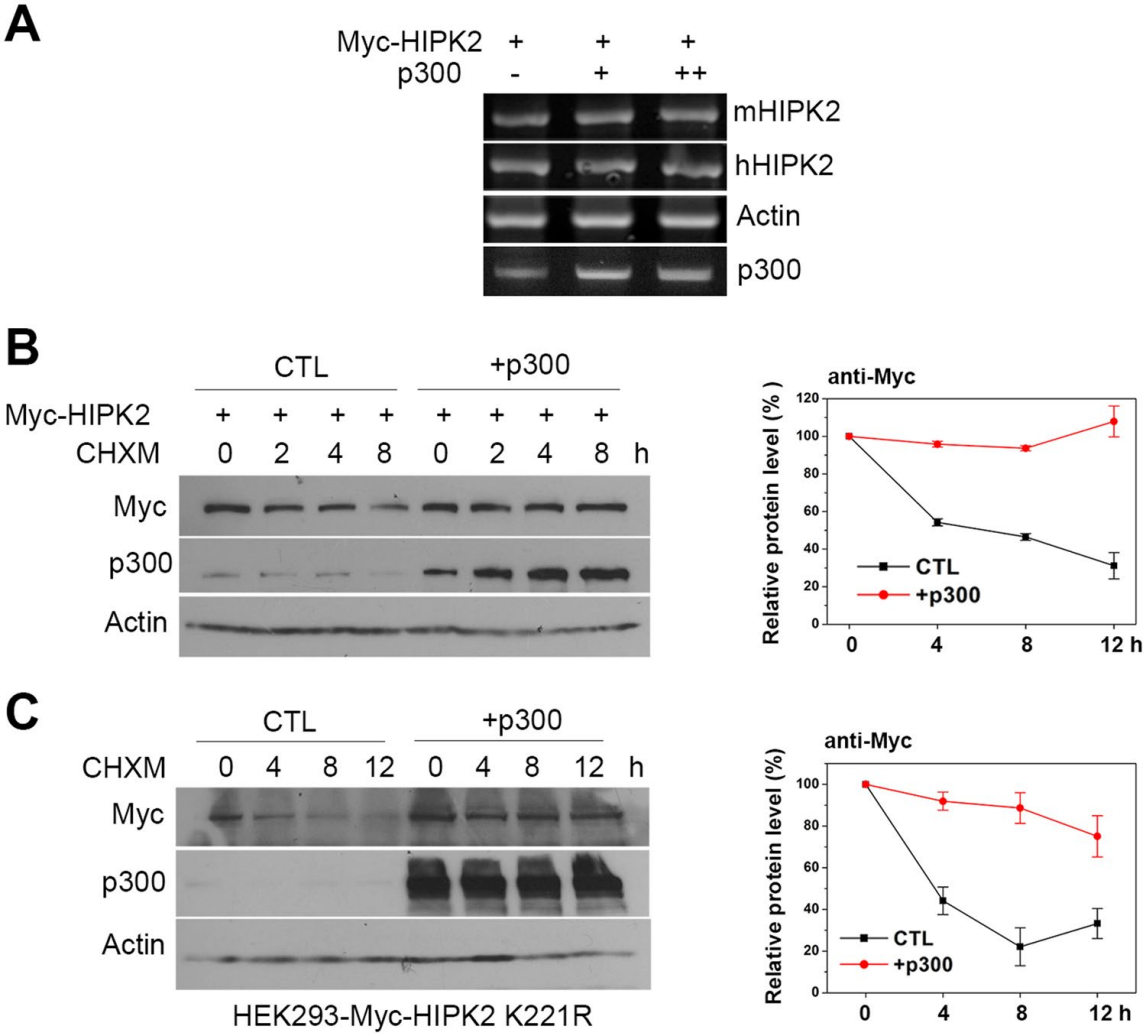
p300 increased the protein stability of HIPK2. (**A**) To examine the mRNA level of HIPK2 when p300 was overexpressed, HEK293 cells were transfected with myc-HIPK2 with or without p300 expression vectors and then processed for RT-PCR using primer pairs for the exogenous mouse (mHIPK2), endogenous human (hHIPK2) HIPK2, actin and p300 at 24 h after the transfection. (**B** and **C**) To examine the protein stability of HIPK2 when p300 was overexpressed, cycloheximide (CHXM, 20 μM) was added into the HEK293 cells transfected with myc-HIPK2 with (+p300) or without p300 (CTL, control) expression plasmids (**B**) or into HEK293 cells stably expressing low level of K221R myc-HIPK2 (**C**) for the indicated times. The cell lysates were processed for the immunoblots using anti-myc and anti-p300 antibodies. Relative protein amounts were quantified by densitometric reading of the immunoblot bands (right panels; means ± SD, n = 3).

Since we observed the upregulation of HIPK2 expression by p300, we then wanted to find out at which level p300 alters the HIPK2 expression. To examine whether p300 can regulate the transcription of HIPK2, mRNA level of HIPK2 was monitored after overexpression of p300. As shown in Fig. 3A, overexpression of p300 did not change the mRNA level of both endogenous and exogenous HIPK2. This result suggests that p300 upregulates the HIPK2 expression at a post-transcriptional level.

The cellular level of HIPK2 protein is known to be regulated tightly by ubiquitin-mediated proteasomal degradation^1^. In addition, we observed that SIRT1 deacetylase decreased the protein stability of HIPK2 by promoting ubiquitination^13^. Therefore, it is possible that the p300-mediated post-transcriptional upregulation of HIPK2 may be due to an increase in the protein stability of HIPK2. To test this possibility, changes in the protein amount of HIPK2 with or without p300 overexpression was monitored in the presence of protein synthesis inhibitor cycloheximide. The protein amount of HIPK2 decreased over 8 h-treatment of cycloheximide but it maintained its initial level when p300 was co-transfected, indicating that p300 increased the protein half-life of HIPK2 (Fig. 3B). In addition, overexpression of p300 also increased the protein stability of the low level exogenous HIPK2 in the HEK293 cells stably expressing kinase-dead myc-HIPK2 (K221R mutant, Fig. 3C). These results indicate that p300 can increase the protein stability of HIPK2.

### p300 promoted acetylation of HIPK2 while decreased its ubiquitination

**Figure 4:**
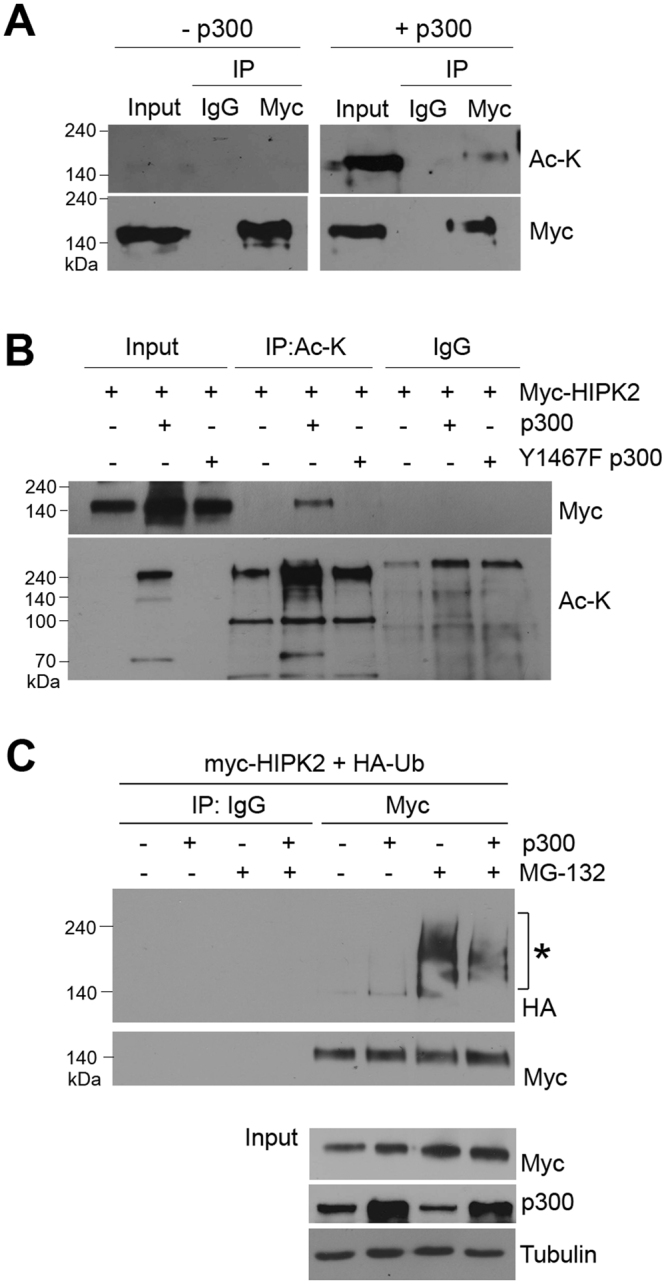
p300 promoted acetylation of HIPK2 while decreased its ubiquitination. (**A**) HEK293 cells were transfected with myc-HIPK2 with or without p300 expression vectors and then immunoprecipitated (IP) using anti-myc antibodies. The immunoprecipitates were then examined by immunoblotting using anti-acetyl lysine (AcK) antibody. (**B**) The cells were transfected with myc-HIPK2 with wild type p300 or Y1467F active site mutant p300 expression vectors and then processed for immunoprecipitation using anti-AcK and immunoblot using anti-myc antibody. (**C**) To monitor the ubiquitination of HIPK2 when p300 was overexpressed, HEK293 cells were transfected with myc-HIPK2 and HA-ubiquitin (HA-Ub) with or without p300 expression vectors and then incubated with MG132 (5 μM) for 10 h. The cells were immunoprecipitated using anti-myc antibody and then immunoblotted using anti-HA antibody. Immunoprecipitation using normal immunoglobulin (IgG) served as a control (**A–C**).

We observed that p300 increased the protein level of HIPK2 via its acetyltransferase activity (Fig. 2). Because the increase of the HIPK2 protein level was due to the increase in protein stability (Fig. 3), it is possible that p300-mediated acetylation on HIPK2 blocks its ubiquitination. To test this possibility, we first examined whether p300 can acetylate HIPK2. When we immunoprecipitated HIPK2 and then immunoblotted using anti-acetyl lysine antibody, the HIPK2 precipitates were reactive with anti-acetyl lysine antibody when p300 was overexpressed (Fig. 4A), suggesting that p300 overexpression promoted the acetylation of HIPK2. This was confirmed when the immunoprecipitation and immunoblot antibodies were switched (Fig. 4B). However, when the active site mutant p300 was overexpressed together with myc-HIPK2, acetylation of the exogenous HIPK2 was not detected (Fig. 4B). These results confirm that p300 acetylates HIPK2.

Then we wanted to determine whether p300 can suppress HIPK2 ubiquitination. We examined the ubiquitination of HIPK2 by immunoblotting for the exogenous ubiquitin after immunoprecipitaing the HIPK2. As shown in Fig. 4C, upwardly shifted bands corresponding to the polyubiquitinated HIPK2 were decreased when p300 was overexpressed. These results suggest that p300 can inhibit the ubiquitination on HIPK2.

### p300 promoted the tumor suppressor function of HIPK2

**Figure 5:**
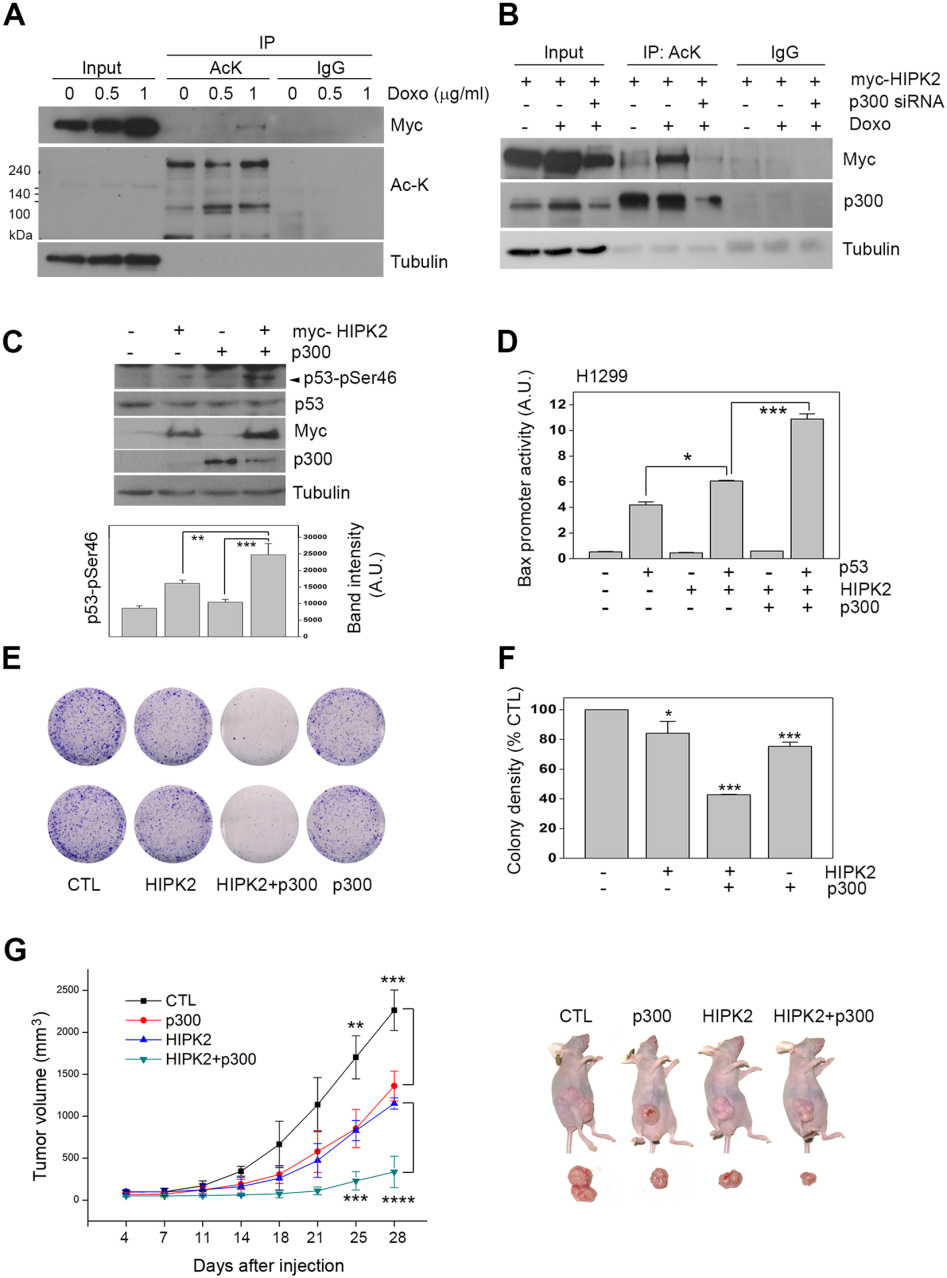
p300 enhanced the tumor suppressor function of HIPK2. (**A**) To examine the acetylation status of HIPK2 under DNA damage condition, HEK293 cells were transfected with expression vectors for myc-HIPK2 and then incubated with increasing concentrations of doxorubicin (Doxo; 0, 0.5, 1 μg/ml) for 6 h. The cell lysates were processed for immunoprecipitation using anti-acetyl lysine (AcK) antibody and then immunoblot using anti-myc antibody. (**B**) To examine the doxorubicin-induced HIPK2 acetylation was mediated by p300, HEK293 cells were transfected with myc-HIPK2 with or without p300 siRNA and then incubated in the presence of doxorubicin for 6 hr (1 μg/ml). The cell lysates were then immunoprecipitated and blotted as in A. (**C**) HEK293 cells overexpressing myc-HIPK2 and/or p300 were processed for immunoblotting using p53 and p53 serine 46 phospho-specific antibodies (p53-pSer46). Tubulin blot served as a loading control. Band intensities of serine 46-phosphorylated p53 were quantified by densitometry and shown in the lower panel (means ± SD, n = 3; **p < 0.01, ***p < 0.001). (**D**) H1299 cells were transfected with p53, myc-HIPK2, or p300 expression vectors plus Bax promoter-luciferase and pRL-TK plasmids and then dual luciferase activity assay was performed. Means ± SD are shown (n = 4; *p < 0.05, ***p < 0.001). (**E** and **F**) HCT116 cells were transfected with empty Flag (CTL, control) or Flag-HIPK2 with or without p300 expression vectors and then selected with G418 (1 mg/ml) for 2 wk. (**E**) Surviving colonies were fixed and stained. (**F**) Densitometric readings of the pixels taken from the photos of the stained cell colonies are shown in **F** (n = 3; *p < 0.05, ***p < 0.001). (**G**) Balb/c nude mice were subcutaneously inoculated with 10^7^ HCT116 cells transfected as indicated. Transfection with empty vector served as control (CTL). Tumor volume was measured twice a week using a caliper. Representative images were taken at day 28 (right). Tumor growth measured for 28 d is shown (left, n = 5, **p < 0.001; ****p < 0.0001).

To assess the functional outcomes of the p300-mediated acetylation and increased stability of HIPK2, we first examined if the acetylation of HIPK2 increases under DNA damage conditions. When the cells were incubated by DNA damaging reagent doxorubicin, both HIPK2 acetylation and its protein level increased (Fig. 5A). To examine if the increased acetylation following doxorubicin treatment was indeed mediated by p300, the doxorubicin-induced acetylation on HIPK2 was monitored after p300 siRNA addition. The downregulation of p300 abrogated the doxorubicin-induced p300 acetylation (Fig. 5B). These results suggest that p300 promotes acetylation of HIPK2 following DNA damage.

We then investigated whether the p300-mediated stabilization of HIPK2 enhances the proapoptotic function of HIPK2. It is well documented that the activation of HIPK2 results in the phosphorylation of p53 at serine 46^21^. Thus we first examined whether p300 can promote HIPK2-mediated phosphorylation of p53. Figure 5C shows that overexpression of HIPK2 increased the phosphorylation of p53 at serine 46 and this was enhanced by p300 co-expression. However, overexpression of p300 alone did not increase the p53 phosphorylation. In addition, when we examined the promoter activity of a p53-target gene Bax^3^, coexpression of p300 enhanced the HIPK2-induced activation of Bax promoter (Fig. 5D). These results suggest that p300 can potentiate the proapoptotic activity of HIPK2.

Then we examined whether p300-mediated enhancement of HIPK2 activation results in the suppression of cell viability. As shown in Fig. 5E and quantified in Fig. 5F, expression of HIPK2 or p300 alone reduced the viability of the cells to a moderate degree but coexpression of the two genes enhanced the viability suppression to a significant degree. This suggests that p300 can enhance the proapoptotic or cell cycle-inhibiting function of HIPK2.

To study whether the p300-mediated promotion of HIPK2 function can operate *in vivo*, we examined the effect of p300 and HIPK2 coexpression in the xenograft tumor model. Immune-deficient nude mice were injected with HCT116 cells overexpressing p300, HIPK2, or p300 plus HIPK2 and then tumor growth was monitored. In accordance with the cell culture data, coexpression of p300 and HIPK2 suppressed the tumor growth much more efficiently than each single gene overexpression. These results suggest that p300 can enhance the tumor suppressor function of HIPK2.

## Discussion

In the present study, we have shown that p300 acetylates HIPK2 and increases its protein stability. Our results suggest that the p300-mediated acetylation suppresses ubiquitination and subsequent proteasomal degradation of HIPK2. We also found that DNA damage can induce the p300-mediated acetylation of HIPK2, which may enhance the proapoptotic function of HIPK2. We also confirmed that p300 can potentiate the tumor suppressor function of HIPK2 in the xenograft tumor model in mice. Previously we showed that HIPK2 is deacetylated by SIRT1 and this promotes ubiquitination and proteasomal degradation of HIPK2^13^. Our previous and present studies first demonstrate that the protein stability of HIPK2 and its tumor suppressor function can be regulated by acetylation.

Protein stability of HIPK2 is tightly regulated by ubiquitination and subsequent proteasomal degradation^8^. Ubiquitin ligases such as Siah1, Siah2, WSB1 and SCF^Fbx3^ have been identified to promote HIPK2 degradation^22^. In unstressed cells, Siah1, WSB1 and SCF^Fbx3^ keep the HIPK2 level low by promoting continuous ubiquitin-mediated proteasomal degradation^22^. It has been reported that the SCF^Fbx3^-mediated degradation of HIPK2 is inhibited by promyelocytic leukemia (PML)^11^. However, the detailed mechanism how PML protects HIPK2 from the SCF^Fbx3^-mediated degradation remains unknown. The degradation of HIPK2 induced by Siah1 or WSB1 was shown to be suppressed upon DNA damage^10,12^. In the case of Siah1, the interaction with HIPK2 seemed to be inhibited by ATM kinase-induced phosphorylation on Siah1^12^. Because the regulation of HIPK2 protein stability under normal vs. stressed conditions can determine the cell fate between survival and death, the regulation of HIPK2 ubiquitination is a matter of great importance. However, much remains to be elucidated how HIPK2 ubiquitination is regulated.

We have shown that acetylation can be a mechanism by which HIPK2 can escape from the continuous proteasomal degradation. One of the possible mechanisms how acetylation can suppress ubiquitination of HIPK2 can be a competition between acetyltransferases and ubiquitin ligases for the critical lysine residues. Examples of such competition between ubiquitin ligases and acetyltransferases on common lysine residues can be found in proteins such as Foxp3 transcription factor and tau^14,18^. In a similar way to our observation on HIPK2, the proteasomal degradation of Foxp3 and tau has been shown to be reciprocally regulated by p300 and SIRT1^14,18^. However, we cannot rule out the possibility that p300-mediated acetylation on HIPK2 alters protein interaction or localization of HIPK2 required for its ubiquitination instead of directly blocking the ubiquitination sites.

Another example of acetylation-mediated regulation of protein stability can be found in p53. Ample evidence suggests that acetylation induces activation as well as stabilization of p53^23^. Extensive studies have revealed that many HATs such as p300/CBP, p300/CBP-associated factor (PCAF), hMOF and TIP60 acetylate p53 on different lysines to induce differential activation of p53 in addition to protein stability change^23^. It remains to be studied whether acetylation on HIPK2 affects its kinase activity apart from its stability. We observed that p300 overexpression resulted in both stabilization and enhancement of tumor suppressor function of HIPK2. Therefore, the possibility still remains that the p300-induced acetylation of HIPK2 fine-tunes it kinase activity as well.

We observed that DNA damaging insult such as doxorubicin treatment promoted the acetylation of HIPK2, which was suppressed by p300 knockdown. This suggests a mechanism by which HIPK2 can accumulate under DNA damage conditions: namely, suppression of ubiquitination by acetylation. As mentioned, driving off the ubiquitin ligases by phosphorylation has been suggested as a mechanism for HIPK2 accumulation. Our present study reveals a different regulation mechanism for HIPK2 stabilization following DNA damage. It has been well documented that p300 is activated by DNA damage^24,25^ and we observed that HIPK2 acetylation following DNA damaging insult was abrogated by p300 knockdown (Fig. 5B). These suggest that HIPK2 is indeed acetylated to be stabilized during DNA damage. It has been also reported that p300 acetylates and activates p53 in response to DNA damage^24,25^. Since HIPK2 activates p53 by phosphorylation^2,3^ and HIPK2 is stabilized by p300 as we have shown in this study, p300 seems to ensure activation of p53 by directly modifying p53 and also stabilizing its upstream regulator HIPK2 under DNA damage conditions.

In conclusion, our study has revealed a mechanism by which HIPK2 protein stability can be regulated. We suggest that p300 suppresses the ubiquitination of HIPK2 by acetylation, increasing the protein stability of HIPK2 to enhance the tumor suppressor function under DNA damage conditions. Increasing HIPK2 stability by promoting its acetylation might be potentially useful to increase the efficacy of chemotherapy targeting HIPK2 or p53.

## Materials and Methods

### Reagents

Epigallocatechin-3-gallate (EGCG) was purchased from Cayman chemical. N-[4-Chloro-3-(trifluoromethyl)phenyl]-2-ethoxy-6-pentadecylbenzamide (CTPB) was from Enzo Life Sciences (Farmingdale, NY, USA). All other reagents were purchased from Sigma (St. Louis, MO, USA) unless stated otherwise.

### Cell culture and viability assay

HEK293 cells were grown in Dulbecco’s modified Eagle’s medium (Welgene, Daegu, Korea) supplemented with 10% heat-inactivated fetal bovine serum and 1% antibiotics plus antimycotics solution (Welgene, Daegu, Korea). Viability of the cells was measured using CellTiter 96 AQueous One Solution assay kit (Promega, Madison, WI, USA).

### Plasmids and transfection

Construction of the expression plasmids for full-length myc-HIPK2, GFP-HIPK2 and various HIPK2 deletion mutants was described previously^26^. Transfection of HEK293 cells with expression vectors encoding p300 or HIPK2 was carried out using Mirus reagent according to the manufacturer’s protocol (Mirus Bio, Madison, WI, USA). For the generation of stable cell line expressing low level of myc-HIPK2, HEK293 cells were transfected with expression plasmid encoding kinase-dead mutant HIPK2 (K221R)^2^, tagged with myc. The transfected cells were selected with G418 (0.2 ~ 1 mg/ml) and cultured for 4 weeks.

### Antibodies and immunostaining

Antibodies used for the immunoblot and immunostaining were anti-p300 (BD Biosciences, San Jose, CA, USA), anti-α-tubulin (Sigma), anti-myc, anti-Flag, anti-GFP, anti-HA (abm, Richmond, Canada), anti-ubiquitin (Millipore, Billerica, MA, USA), anti-acetylated lysine (Cell signaling, Danvers, MA, USA). Anti-HIPK2 antibody was a kind gift of Dr. K. Isono (RIKEN, Japan) and described previously^27^.

### Immunoprecipitation and immunoblot

Immunoprecipitation was performed using 2 × 10^7^ cells in a lysis buffer (20 mM Hepes, pH 7.5, 0.1 M KCl, 0.4 mM EDTA, 0.2% Nonidet P-40, 10 mM β-mercaptoethanol, 1 μg/ml sodium vanadate, 10 μg/ml leupeptin, 10 μg/ml aprotinin, 0.1 mM PMSF). After incubation at 4 °C on a rotating wheel for 15 min and centrifugation at 20,000 × g for 10 min at 4 °C, equal volumes of protein were diluted with lysis buffer lacking NaCl and KCl, then incubated overnight with antibodies. Then protein A sepharose beads were added (Sigma). After incubation at 4 °C for 1 h with agitation, the beads were washed three times with lysis buffer. Immunoblotting was performed by conventional methods. Original images of uncropped blots are shown in the supplementary material.

### Reverse transcription polymerase chain reaction

Total RNA was isolated using RNeasy minikit (Qiagen, Valenia, CA). cDNA was synthesized using the Moloney Murine Leukemia Virus Reverse Transcriptase (Promega, Madison, WI, USA) according to the manufacturer’s protocol. Reverse transcription reaction product was used as a template for PCR using the following primer pairs: mouse HIPK2 (forward 5′-GTC ACC ATG ACA CAC CTG CT-3′, reverse 5′-AGG GGG ACA CAC GAT GAG AG-3′), human HIPK2 (forward 5′-CCA CAG CAC ACA CGT CAA ATC-3′, reverse 5′-TTT GCT CTG GTT CAC CGT GTC-3′), human p300 forward 5′-GCC ACC ATG GAG AAG CAT AA-3′, reverse 5′-AGA TCG CAG GGG ATG GAG-3′), β-actin (forward 5′-CTG GGA CGA CAT GGA GAA-3′, reverse 5′-AAG GAA GGC TGG AAG AGT-3′). Annealing temperature was 58 °C. Reaction products were analyzed on 2% agarose gels.

### Luciferase assay

To measure Bax promoter activity, H1299 cells were transfected with expression vectors encoding p53, p300 and HIPK2, along with the bax-luciferase reporter vector and control pRL-TK plasmids. At 24 h after the transfection, dual luciferase activity assay was performed using dual luciferase reporter assay kit (Promega) according to the manufacturer’s protocol.

### Cell colony formation assay

HIPK2 expression vector (pCMV-Flag) was transfected into HCT116 cells, with or without p300 expression vector. At 24 h after the transfection, cells were selected with G418 (1 mg/ml) for 2 weeks. Surviving colonies were fixed with 4% paraformaldehyde and stained with crystal violet.

### Xenograft tumor model

Animal use complied with the National Institutes of Health Guide for the Care and Use of Laboratory Animals and was approved by the Institutional Animal Care and Use Committee at Sejong University. Five-week-old immune-deficient BalB/c nude mice (Nara Biotech, Seoul, Korea) were group housed in pressurized ventilated cages under a 12:12 light-dark lighting schedule with free access to food and water in an SPF facility. Transfected HCT116 cells (1 × 10^7^) were injected into mice subcutaneously. Tumor growth was monitored periodically and measured using calipers. Tumor volumes were calculated by the formula: Tumor volume = width^2^ × length × 1/2. The weight of the tumor was measured after sacrificing the animal.

### Statistics

For the statistical analysis, all the experiments were repeated at least three times. The results were expressed as means ± SD of three independent experiments, unless stated otherwise. Statistical significance of the data was evaluated by student’s *t*-test.

## Electronic supplementary material

Supplementary information
