# Supplementary material for: p300-mediated acetylation increased the protein stability of HIPK2 and enhanced its tumor suppressor function
Source: Sci Rep. 2017 Nov 23;7:16136. doi: 10.1038/s41598-017-16489-w (PMC5701035; doi:10.1038/s41598-017-16489-w)
Supplement: Supplementary file 1 — Supplementary information [file 41598_2017_16489_MOESM1_ESM.pdf]

p300-mediated acetylation increased the protein stability of HIPK2 and enhanced its tumor suppressor function

Jong-Ryoul Choi[1*#], Seo-Young Lee[1*], Ki Soon Shin[2, 3], Cheol Yong Choi[4], Shin Jung Kang[1, 5]

1 Department of Molecular Biology, Sejong University, Seoul 05006, Republic of Korea

2 Department of Biology, Kyung Hee University, Seoul 02447, Republic of Korea

3 Department of Life and Nanopharmaceutical Sciences, Kyung Hee University, Seoul 02447, Republic of Korea

4 Department of Biological Sciences, Sungkyunkwan University, 2066 Seobu-ro, Suwon 16419, Republic of Korea

5 Department of Integrative Bioscience and Biotechnology, Sejong University, Seoul 05006, Republic of Korea

* These authors equally contributed to this work.

Corresponding author: Shin Jung Kang

Tel: 82-2-3408-3943

Fax: 82-2-3408-4336

Email: sjkang@sejong.ac.kr

Address: Department of Molecular Biology, Sejong University, 209 Neungdong-ro, Gwangjin-gu, Seoul 05006, Korea

#Current address: Massachusetts General Hospital, Cutaneous Biology Research Center, Building 149, 13th Street, Charlestown, MA 02129

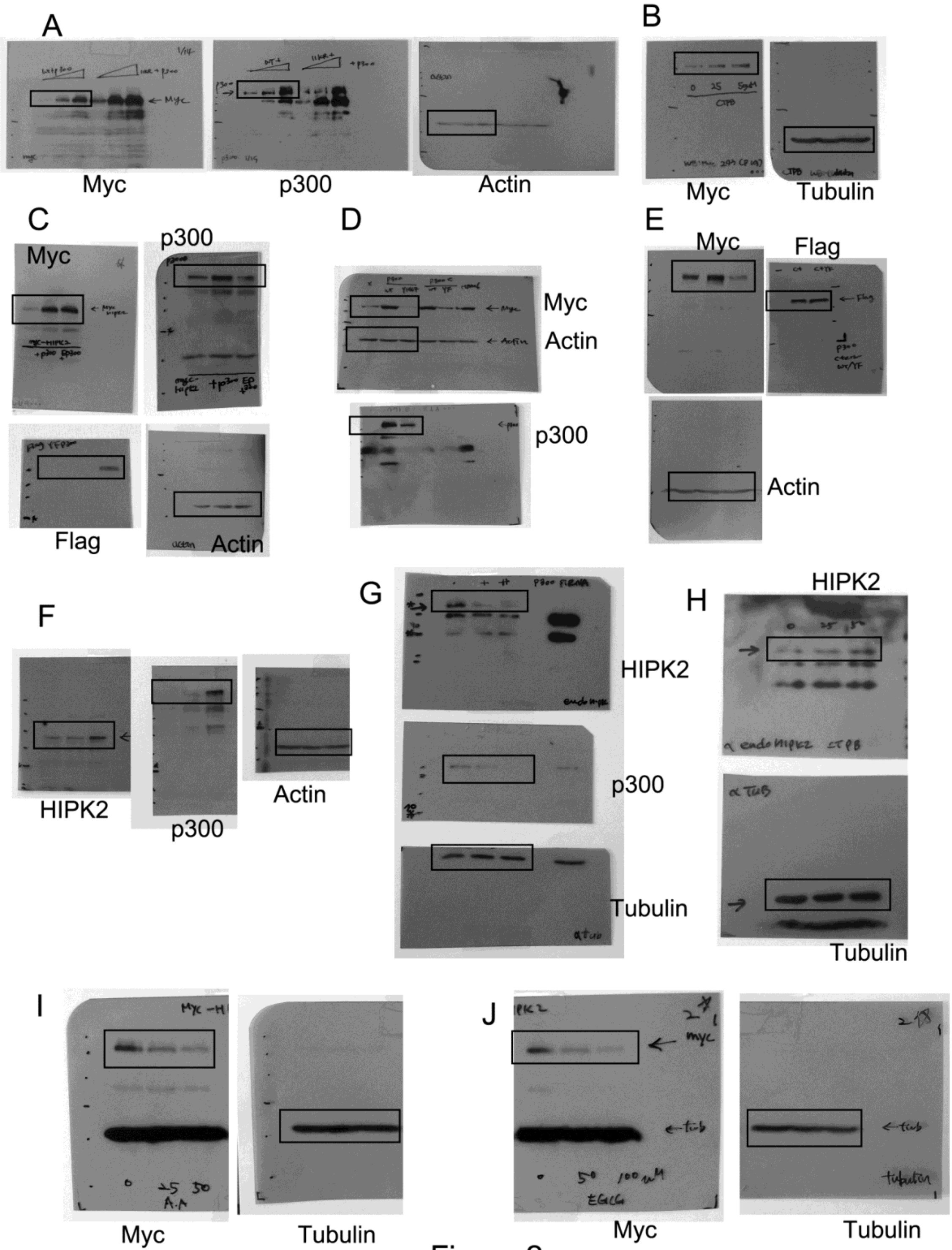

A
Myc
p300
Actin
B
Myc
Tubulin
C
Myc
p300
Flag
Actin
D
Myc
Actin
p300
E
Myc
Flag
Actin
F
HIPK2
p300
Actin
G
HIPK2
p300
Tubulin
H
HIPK2
Tubulin
I
Myc
Tubulin
J
Myc
Tubulin

Figure 2

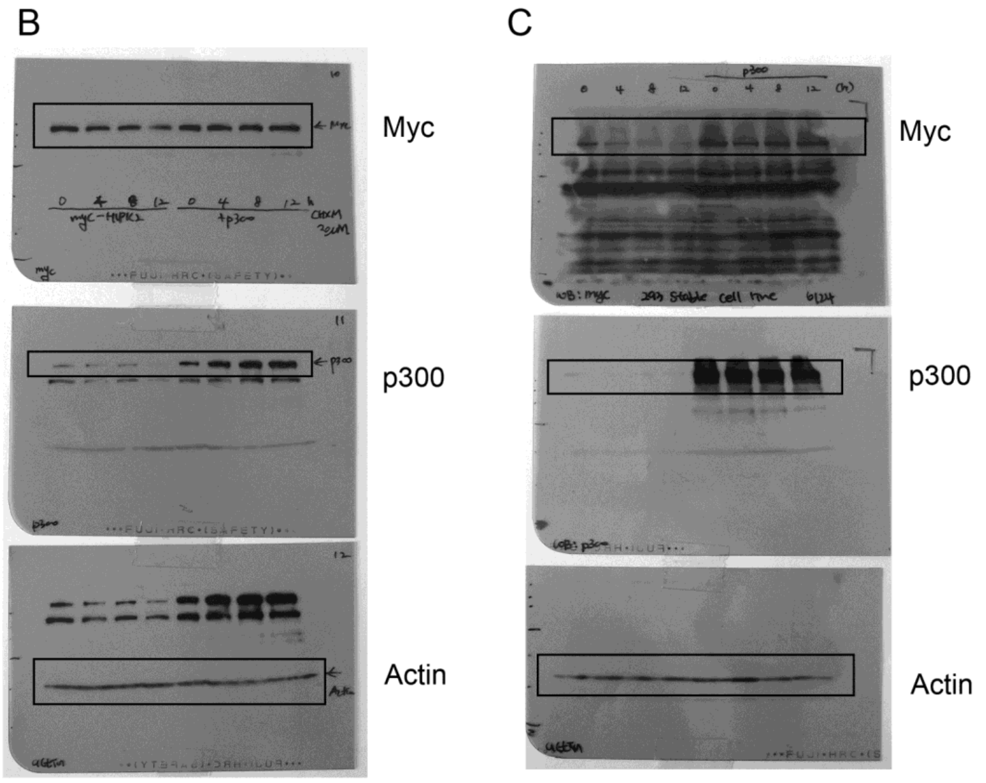

Figure 3

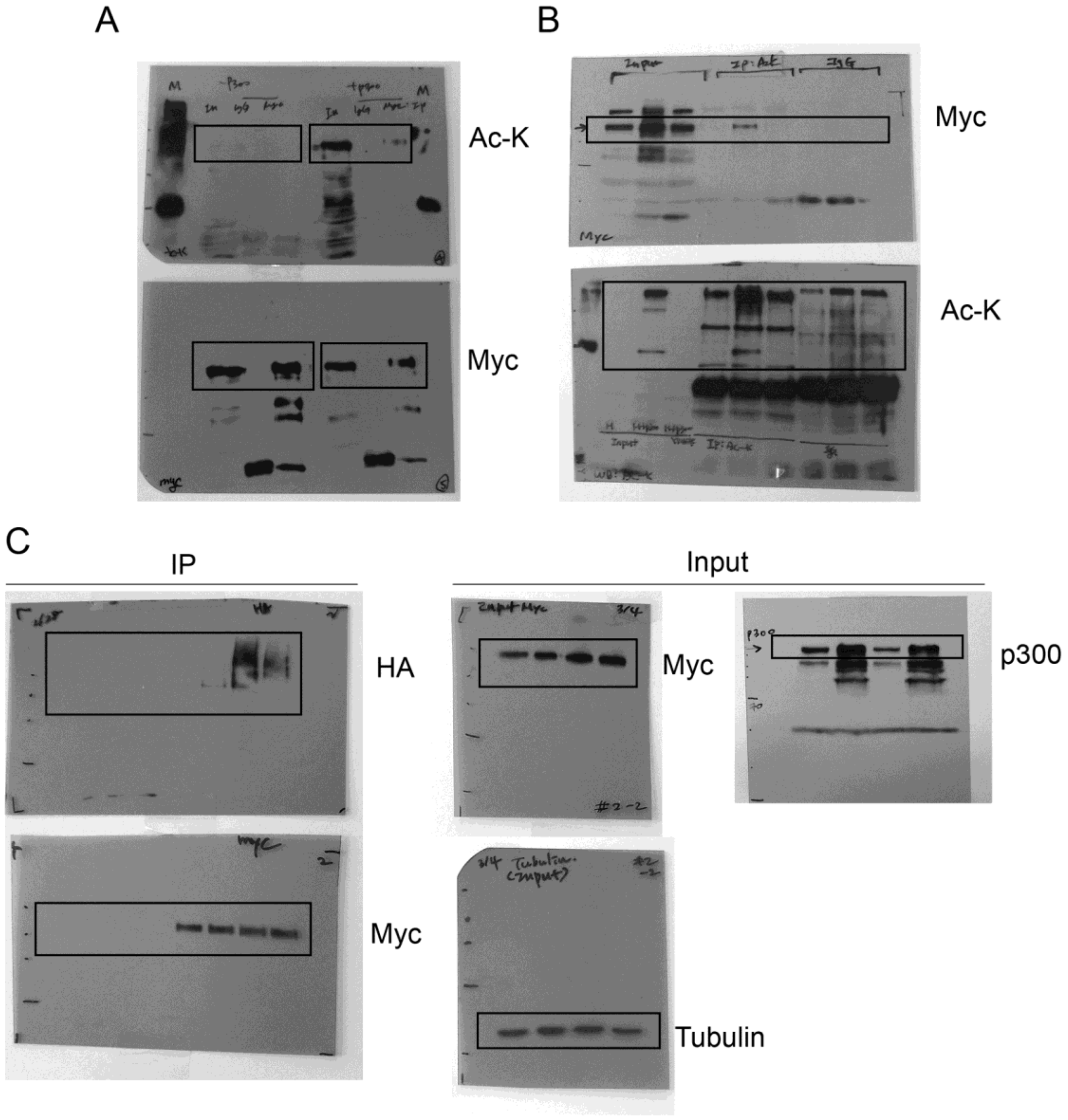

Figure 4

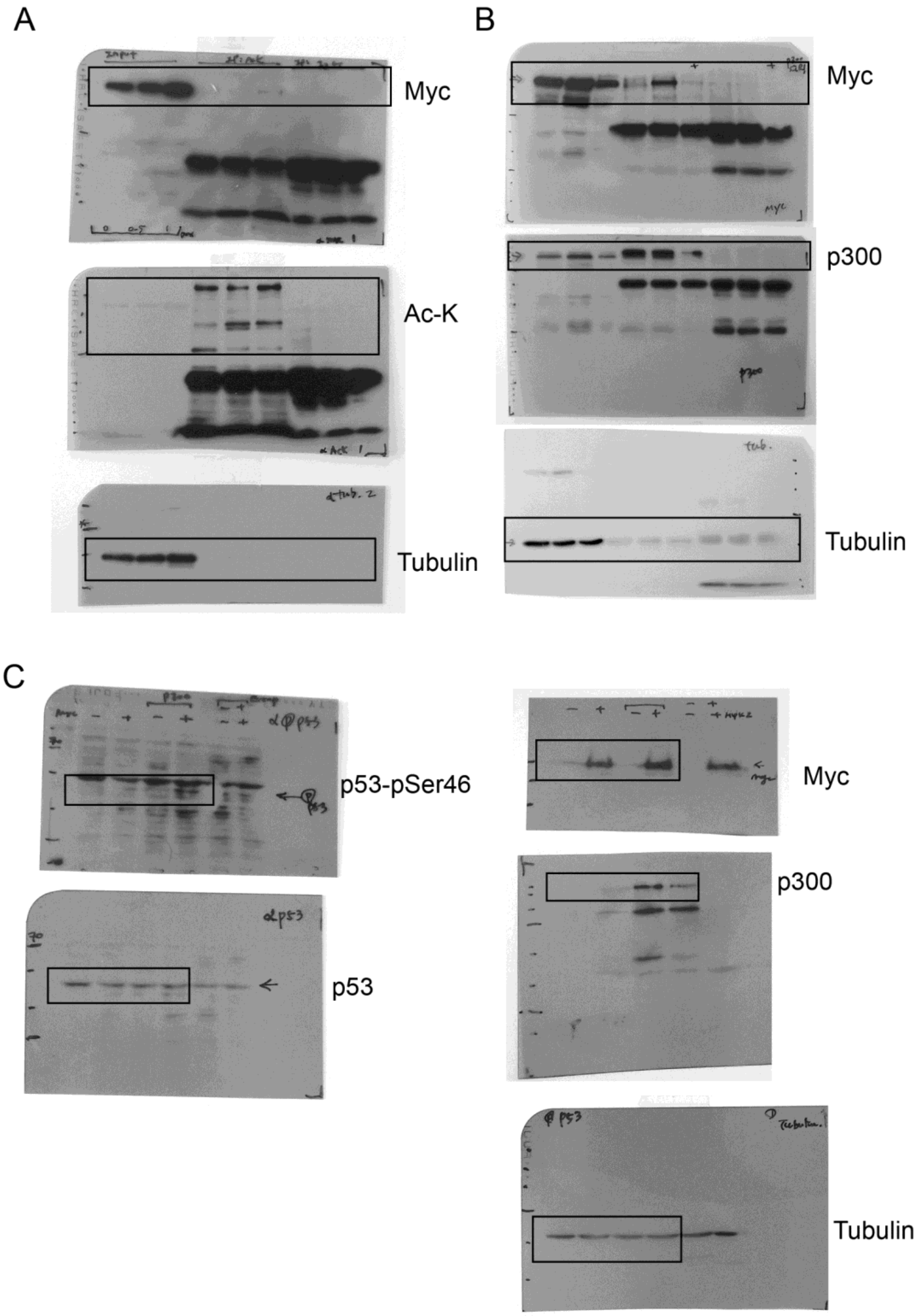
A
Myc
Ac-K
Tubulin
B
Myc
p300
Tubulin
C
p53-pSer46
p53
Myc
p300
Tubulin

Figure 5
